# PSTPIP2 Inhibits the Inflammatory Response and Proliferation of Fibroblast-Like Synoviocytes *in vitro*

**DOI:** 10.3389/fphar.2018.01432

**Published:** 2018-12-04

**Authors:** Yao Yao, Haixia Yu, Yaru Liu, Qingqing Xu, Xiaofeng Li, Xiaoming Meng, Cheng Huang, Jun Li

**Affiliations:** ^1^Anhui Province Key Laboratory of Major Autoimmune Diseases, Anhui Institute of Innovative Drugs, School of Pharmacy, Anhui Medical University, Hefei, China; ^2^The Key Laboratory of Anti-inflammatory and Immune Medicines, Ministry of Education, Hefei, China

**Keywords:** rheumatoid arthritis, PSTPIP2, FLSs, inflammatory response, proliferation, NF-κB signaling pathway

## Abstract

Rheumatoid arthritis (RA) is a chronic autoimmune inflammatory disease and its pathogenesis remains unclear. Fibroblast-like synoviocytes (FLSs) play an important role in the pathogenesis of RA. Proline-serine-threonine phosphatase interacting protein 2 (PSTPIP2) is an adaptor protein, which is associated with auto-inflammatory disease. In this study, we selected adjuvant-induced arthritis (AIA) as animal model to study the role of PSTPIP2 in FLSs. We found that the expression of PSTPIP2 was significantly down-regulated in synovial tissues and FLSs of AIA rat compared with normal group. And overexpression of PSTPIP2 could inhibit the proliferation and inflammatory response of FLSs. Moreover, the proliferation and inflammatory response of FLSs were promoted with PSTPIP2 silencing treatment. In terms of mechanism, we found that the expression of PSTPIP2 was closely related to NF-κB signaling pathway. Overall, our results suggested that PSTPIP2 inhibits the proliferation and inflammatory response of FLSs, which might be closely related to NF-κB signaling pathway.

## Introduction

Rheumatoid arthritis (RA) is a chronic inflammatory and autoimmune disease (Miossec, 2013; Kriegsmann et al., 2016; Mastroleo, 2016). About 30% of RA patients would become permanently work disabled in the first 2–3 years without sufficient and effective treatment (Yang and Chen, 2015). The development of new treatments targeting specific pathophysiological factors would generate crucial understanding toward the complex mechanisms underlying RA, and produce improved outcomes.

Fibroblast-like synoviocytes (FLSs) play an important role in the development of synovitis and joint damage in RA (Miao et al., 2013). Activated FLSs in RA exhibit tumor-like behavior such as invasion, migration, and hyperproliferation, but no similar phenomena are observed in other fibroblasts (Bottini and Firestein, 2013). Hyperproliferative FLSs release large amounts of pro-inflammatory cytokines, leading to destruction of bone and cartilage (Tolboom et al., 2002; Duarte, 2015; Mu et al., 2016; Zou et al., 2016). Therefore, inhibiting the proliferation and inflammatory response of FLSs may be a potentially therapeutic strategy for the treatment of RA.

PSTPIP2, a protein associated with auto-inflammatory disease, has been demonstrated to play a role in innate immunity and auto-inflammatory bone disorders (Drobek et al., 2015; Marzano et al., 2016). Studies established that PSTPIP2 regulates the proliferation and differentiation of megakaryocytes (Liu et al., 2014). In recent years, evidence suggested that PSTPIP2 plays an increasingly important role in inflammatory diseases in addition to their functions in hematopoietic cells. Other studies also indicated that PSTPIP2 is a negative regulator of IL-1β (a key pro-inflammatory cytokine in inflammation) (Lukens et al., 2014). PSTPIP2 has been known for its principal role in the development of inflammatory disorder described as chronic multifocal osteomyelitis in mice, where its reduced expression or complete absence is the main cause of the disease (Ferguson et al., 2006; Grosse et al., 2006; Chitu et al., 2009; Gurung et al., 2016). It was indicated that missense mutation in PSTPIP2 gene results in the development of spontaneous chronic bone disease characterized by bone deformity (Gurung et al., 2016). Moreover, there was direct evidence that PSTPIP2 could suppress inflammatory response (Drobek et al., 2015; Marzano et al., 2016). Nevertheless, the role of PSTPIP2 in RA has not been reported. Therefore, it is of great significance to explore whether PSTPSP2 is related to RA.

We performed vital experiments first and surprisingly found that the expression of PSTPIP2 in synovial tissues and FLSs of AIA rat was aberrant down-regulated compared with normal group. Overexpression of PSTPIP2 was negatively correlated with the proliferation and inflammatory response of FLSs. Interestingly, the proliferation and inflammatory response of FLSs were promoted with PSTPIP2 silencing treatment. Moreover, we found that the NF-κB pathway was related to the expression of PSTPIP2 in FLSs. Collectively, these results suggested that PSTPIP2 inhibits the proliferation and inflammatory response of FLSs in AIA, which might be closely related to the NF-κB signaling pathway.

## Materials and Methods

### Materials and Reagents

Fetal bovine serum (FBS) was acquired from Gibco (United States). High glucose Dulbecco’s modified Eagle’s medium (DMEM) was purchased from Hyclone (Logan, UT, United States). Complete Freund’s Adjuvant (CFA) was obtained from Sigma Chemical (St. Louis, MO, United States). Rabbit anti-PSTPIP2 (bs-19580R) was purchased from Bioss (Beijing, China). Rabbit anti-TNF-α (ab6671), anti-IL-6 (ab9324), and anti-IL-1β (ab9722) were purchased from Abcam (Britain). Rabbit anti-p-P65 (YP0191, phospho Ser536), rabbit anti-Iκ B (YT2419), and rabbit anti-p-IκB (YP0151, phospho Ser32/S36) polyclonal antibody were obtained from ImmunoWay Biotechnology Company (Plano, TX, United States). Rabbit anti-c-Myc and rabbit anti-CyclinD1 monoclonal antibody were purchased from Cell Signaling (Danvers, MA, United States). Rat anti-β-actin monoclonal antibody was purchased from Bioworld (Shanghai, China). Rabbit anti-P65 (EAP1020) was purchased from Elabscience Biotechnology Co., Ltd. (Wuhan, Chain). Rabbit anti-Vimentin monoclonal antibody was purchased from Bioss Biotechnology Co., Ltd. (Beijing, China). PSTPIP2, TNF-α, IL-6, IL-1β, and β-actin primers were compounded by Shanghai Sangon Biological and Technological Company (Shanghai, China). Secondary antibody for goat anti-rabbit immunoglobulin (IgG) horseradish peroxidase (HRP) was purchased from Beijing Zhongshan Biotechnology Corporation (Beijing, China). TNF-α, IL-6, and IL-1β Enzyme Linked Immunosorbent Assay (ELISA) kit were purchased from Elabscience Biotechnology Co., Ltd. (Wuhan, China). Cell Cycle Studies Using kits and cell proliferation and tracer assay kit (CFDA SE) (C0051) were purchased from Beyotime (China).

### RA Rat Model

Animal protocols were approved by the Animal Care and Use Committee of Anhui Medical University, China. Female Sprague-Dawley (SD) rats (160–200 g) were injected with 0.1 ml (5 mg/ml) CFA at the left hind paw on day 0. Normal control rats were injected with phosphate buffer saline (PBS) at the same time. Animals were divided into two groups (*n* = 5 per group) randomly, which are normal group (M) and model group (N). All animals were killed on day 24 after adjuvant injection for further examination.

### Histopathology

The knee tissues of each SD rat’s right hind leg were excised and then perfused in 4% paraformaldehyde for at least 48 h. After fixation, the knee tissues were decalcified and embedded in paraffin, and then they were used for hematoxylin and eosin (H&E) staining and immunohistochemistry analysis by standard protocols. The stained sections of HE were then assessed *via* changes in vasospasm, inflammatory cell infiltration, and synovial hyperplasia.

### Cell Culture

Fibroblast-Like Synoviocytes were derived from the synovial tissues of model group and normal group. The rats were killed on day 24 and the knee joints were promptly removed for subsequent experiments. FLSs were prepared by the method of tissue explant cultivation as described. Fresh synovial membranes obtained from the knee joints were minced, incubated in a culture bottle, and maintained in high glucose DMEM medium with 20% (vol/vol) FBS and Penicillin–Streptomycin Solution (Beyotime, China) at 37°C in 5% CO_2_ for 7 days. After removal of the synovial pieces, the adherent cells were cultured in medium. At 70–80% confluence, adherent cells were trypsinized, separated at a 1:2 ratio, and cultured in medium. After three passages, most of the cultured synoviocytes comprised a homogeneous population of FLSs. In the following experiments, the FLSs of passages 3 were used.

### ELISA Assay

The levels of IL-6, IL-1β, and TNF-α in serum and FLSs supernatant were measured by ELISA Kit according to the manufacturer’s instructions. Optical density values were detected at 450 nm. Three independent experiments were performed and each sample was quantified with three replicates.

### Immunofluorescence Staining

Cultured FLSs were plated in DMEM supplemented with 20% FBS at a density of 1–2 × 10^5^ cells/ml. Immunofluorescence staining was performed with rabbit anti-PSTPIP2 and rabbit anti-Vimentin antibodies. Alexa Fluor 488-Conjugated AffiniPure Goat anti-rabbit IgG (H+L) was used as secondary antibody. Counterstaining of nuclei was performed with 4’,6-diamidino-2-phenylindole (DAPI; Beyotime, China). Stained FLSs were examined with an inverted fluorescence microscope (OLYMPUS IX83, Tokyo, Japan).

### Small RNAi Transfection

FLSs were transfected with 100 nM of small interfering RNA (siRNA) using Lipofectamine 2000 (Invitrogen, CA, United States) according to the manufacturer’s instructions. The PSTPIP2-siRNA (PSTPIP2-RNAi) sense strand is 5’-GCAGUGCCAAUAUGGCCAATT-3’ and antisense strand is 5’-UUGGCCAUAUUGGCACUGCTT-3’. The scrambled-siRNA (NC-RNAi) sense strand is 5’-UUCUCCGAACGUGUCACGUTT-3’, and antisense strand is 5’-ACGUGACACGUUCGGAGAATT-3’. The cells were cultured at 37°C for 6 h, then we changed the opti-MEM by DMEM including 20% FBS, and the cells were cultured for another 48 h. After that, qRT-PCR, Western blot, and flow cytometer were used to detect the related indicators.

### Plasmid Construction and Transfection of FLS

Overexpression plasmid for PSTPIP2 was generated by GENE Corporation (Shanghai, China). The following primers were served as amplification: forward:

5’-GAGGATCCCCGGGTACCGGTCGCCACCATGGCGGAGCCGAGCGGC-3’; reverse: 5’-TCACCATGGTGGCGACCGGGCTGACACTCAIACTGAGCA-3’. FLSs (1 × 10^4^ cells) were cultured in 6-well plates with antibiotics-free DMEM for 24 h, then transfected with GV230-PSTPIP2 or GV230 by using Lipofectamine 2000 reagent (Invitrogen, United States) according to the manufacturer’s instruction. Cell grouping: control, model, GV230, and GV230-PSTPIP2. The cells were cultured at 37°C for 6 h, then we changed the opti-MEM by DMEM including 20% FBS, and the cells were cultured for another 48 h. After that, qRT-PCR, Western blot, and flow cytometer were used to detect the related indicators.

### CFDA SE Cell Proliferation Assay and Tracking Kit

To analyze the effect of the expression of PSTPIP2 on FLSs proliferation, the CFDA SE kit was used (Beyotime, China). Briefly, FLSs were transfected, and then the cells were labeled with CFDA SE at 48 h post-transfection. The cells were detected by flow cytometry at 0 h post-label and 48 h post-label, respectively (Xia et al., 2014). All experiments were performed in triplicate and repeated three times. Since the fluorescence of CFDA SE-labeled cells is very uniform and stable, the fluorescence of each progeny cell is reduced by half. So that cells without division can be detected by flow cytometry, and cells that divide once (1/2 fluorescence intensity), cells that divide twice (1/4 fluorescence intensity), cells that divide thrice (1/8 fluorescence intensity), and similar cells with other divisions. All experiments were performed in triplicate and repeated three times. Cell proliferation was quantified by calculating the distance of the peak of 48 h relative to the right shift of the peak of 0 h.

### Cell Cycle Analysis

To analyze the intracellular DNA content, FLSs were fixed in 70% ethanol at 4°C overnight at 48 h after treatment with PSTPIP2-siRNA or GV230-PSTPIP2. FLSs were centrifuged at 1000 *g* for 5 min and re-suspended in PBS. After then, the cells were stained with 0.5 ml propidium iodide (PI) staining buffer contains 200 mg/ml RNase A and 50 μg/ml PI at room temperature for 30 min in the dark. Then flow cytometry was used to analyze. All experiments were performed in triplicate and repeated three times.

### Real-Time Quantitative PCR

Total RNA was obtained from synovial tissues and FLSs using TRIZOL reagent (Invitrogen, United States) and quantified by the Thermo Scientific NanodROP 2000 Spectrophotometer (Thermo Scientific, United States). Then the cDNA was reversely transcribed using TAKARA (Japan). The reaction was prepared following manufacturer’s protocol using SYBRGreen qPCR Master Mix (TAKARA, Japanese) in Pikoreal 96 Real-time PCR system (Thermo Scientific, United States) to detect the level of mRNA. The primers which we used were as follows: Rat PSTPIP2, 5’-TGCTGCCCACAIAIACAGAGGA-3’ (forward primer), 5’-TATTGGCACTGCGGTGGACAG-3 (reverse primer). Rat β-actin, 5’-CCCATCTATGAGGGTTACGC-3’ (forward primer), 5’-TTTAIATGTCACGCACGATTTC-3’ (reverse primer). Rat TNF-α 5’-ACTCCCAGAIAIAIAGCAIAGCAIA-3’ (forward primer), 5’-CAGTTCCACATCTCGGATCA-3’ (reverse primer). Rat IL-6 5’-GAGCCCACCAGGAIACGAIAIAGTC-3’ (forward primer), 5’-TGTTGTGGGTGGTATCCTCTGTGAIA-3’ (reverse primer). Rat IL-1β 5’-TTGACTTGGGCTGTCCAGAT-3’ (forward primer), 5’-CTCCACAGCCACAIATGAGTG-3’ (reverse primer). The level of endogenous β-actin mRNA was served as an internal control and the relative level of mRNA was analyzed by the 2^–ΔΔ^ method. The first-strand cDNA was synthesized by using Prime Script RT Reagent kit (Takara, JAP) according to the manufacturer’s instruction. The reverse transcription condition: 37°C for 15 min, 85°C for 5 s, and 37°C for 10 min. qRT-PCR was performed at 95°C for 10 min followed by 40 cycles at 95°C for 15 s and at 60°C for about 1 min by using Thermo Step One.

### Western Blot

Synovial tissues and FLSs were lysed by Radio-Immunoprecipitation Assay (RIPA) reagent containing 1% phenylmethanesulfonylfluoride (Beyotime, China). Protein concentration of the extractive was quantified by the Thermo Scientific NanodROP 2000 Spectrophotometer (Thermo Scientific, United States). Equal amounts of protein were electrophoresed to sodium dodecyl sulfate polyacrylamide gel and blotted onto Poly(vinylidene fluoride) membranes (Millipore Corp., Billerica, MA, United States). After blockade with 5% milk, nitrocellulose blots were incubated with primary antibodies diluted with primary antibody dilution buffer (Beyotime, China). The primary antibodies recognizing PSTPIP2 (1:500), TNF-α (1:1000), IL-6 (1:1000), IL-1β (1:1000), P65 (1:1000), p-P65 (1:1000), IκB (1:1000) and p-IκB (1:1000), c-Myc (1:500), CyclinD1 (1:500), and β-actin (1:1000) were used respectively. After 12 or 24 h, the membranes were washed three times with TBS/Tween 20 (0.075%) (TBST), and then the blots were incubated with secondary antibodies (1:8000) for 1 h including goat anti-rat or goat anti-rabbit horseradish peroxidase (HRP, Zhong-shan Biotechnology Corporation, Beijing, China) in TBST containing 5% skim milk. The membranes were washed for three times with TBST. At last, the protein bands were analyzed by ECL-chemiluminescence kit (ECL-plus, Thermo Scientific, United States). The variation in the density of bands was presented as fold changes compared with the control after normalized to β-actin.

### Rat Arthritis Scores and Paw Swelling Score

Animals were placed individually in transparent beaker first and then rat arthritis score was evaluated according to the following rules: severity of redness (0–normal, 1–slightly red/purple, 2–red/purple), degree of swelling (0–normal, 1–slight swelling at the injection site, 2–swelling at the injection site and toes or ankle, 3–swelling at the injection site, toes, and ankle), and variation of claw (0–normal, 1–slightly curved, 2–curved, 3–almost closed) (Rossato et al., 2015). Right hind paw swelling was measured by the paw volume meter at the 12th, 14th, 16th, 18th, 20th, and 22th days. Paw swelling score is the difference between the paw volume of the 12th, 14th, 16th, 18th, 20th, and 22th days and paw volume without injecting CFA at the first day. Each group contains 5 rats.

### Statistical Analysis

Data are presented as the mean ± SD, and the data were acquired from at least three experiments. Statistical significance was determined by one-way or two analysis of variance (ANOVA) followed by Tukey’s HSD test, when appropriate. *P* < 0.05 was defined as statistically significant.

## Results

### PSTPIP2 Was Significantly Down-Regulated in Synovial Tissues and FLSs of AIA

**FIGURE 1:**
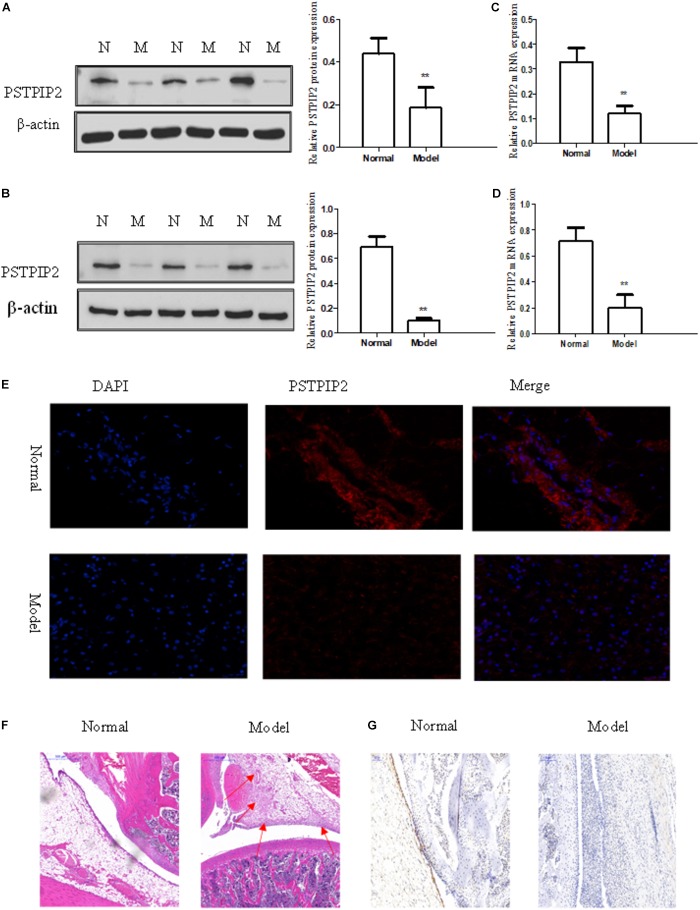
PSTPIP2 was significantly down-regulated in synovial tissues and FLSs of AIA. **(A)** The protein level of PSTPIP2 was analyzed by Western blot in AIA and normal synovial tissues. **(B)** The protein level of PSTPIP2 was analyzed by Western blot in AIA and normal FLSs. **(C)** The mRNA level of PSTPIP2 was analyzed by qRT-PCR in AIA and normal synovial tissues. **(D)** The mRNA level of PSTPIP2 was analyzed by qRT-PCR in AIA and normal FLSs. **(E)** The expression of PSTPIP2 was analyzed by immunofluorescence staining analysis in FLSs. **(F)** Representative H&E staining of AIA and normal synovial tissues in rat. **(G)** The expression of PSTPIP2 in synovial tissue was analyzed by IHC staining analysis. The bands or images in the figure are representative in three independent experiments. Each group contains 5 rats. Data shown are the mean ± SD from three independent experiments. ^∗∗^*P* < 0.01 versus normal group.

The model of AIA was established by injection of CFA, and typical photos of non-injected hind paws (right hind paw) were taken (Supplementary Figure S1A). Rat arthritis score was evaluated (Supplementary Figure S1B) and paw swelling score was measured (Supplementary Figure S1D). Each group contains 5 rats. Serological test was performed to detect the levels of IL-6, IL-1β, and TNF-α in serum obtained from rats by ELISA kits (Supplementary Figure S1C). Histopathological analysis (Figure 1F) confirmed that the model of AIA was established successfully, increasing remarkable inflammatory cell infiltration, vasospasm, and synovial hyperplasia were observed in AIA knee tissues compared with normal group. The expression of Vimentin (Supplementary Figure S1E) indicated that the cells derived from synovial tissues were FLSs. Western blot results showed that the protein level of PSTPIP2 was significantly reduced in AIA rat synovial tissues (Figure 1A) and FLSs (Figure 1B) compared with normal rats. qRT-PCR data suggested that the mRNA level of PSTPIP2 was reduced in AIA rat synovial tissues (Figure 1C) and FLSs (Figure 1D) compared with normal rats. The immunohistochemical analysis showed that the expression of PSTPIP2 was down-regulated in AIA rat synovial tissues (Figure 1G). And immunofluorescence assay also suggested that the expression of PSTPIP2 was reduced in AIA rat FLSs (Figure 1E). These results indicated that PSTPIP2 is significantly down-regulated in AIA rat synovial tissues and FLSs compared with normal rats.

### Overexpression of PSTPIP2 Suppresses FLSs Inflammatory Response

**FIGURE 2:**
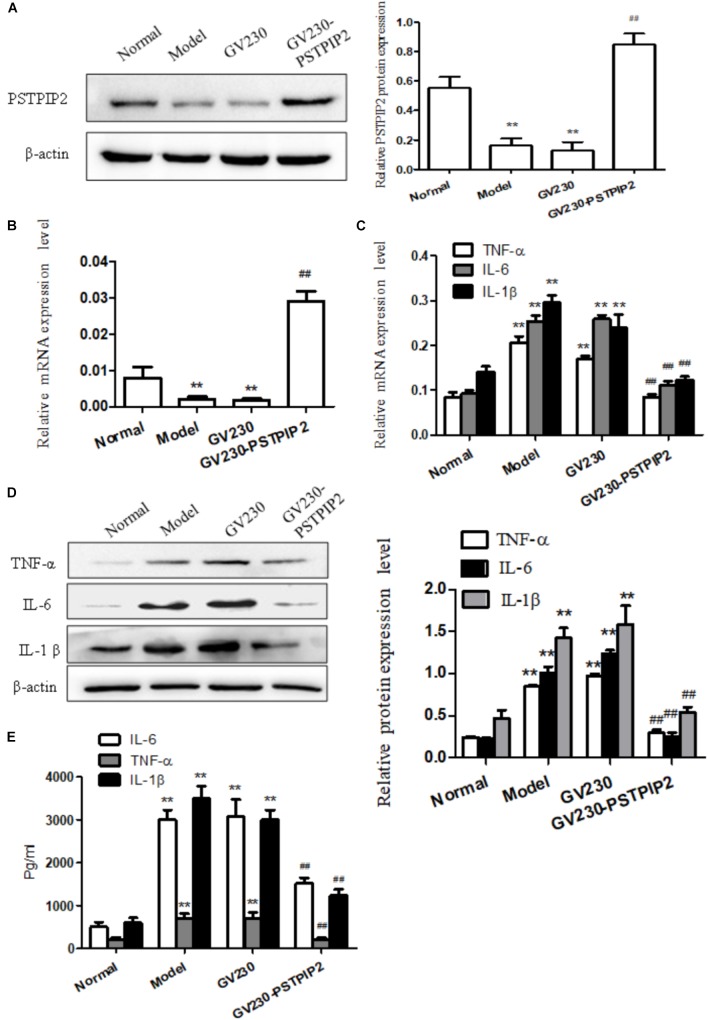
Overexpression of PSTPIP2 suppresses FLSs inflammatory response. **(A)** The protein level of PSTPIP2 was analyzed by Western blot in FLSs with PSTPIP2 overexpression. **(B)** The mRNA level of PSTPIP2 was analyzed by qRT-PCR in FLSs with PSTPIP2 overexpression. **(C)** The mRNA levels of TNF-α, IL-1β, and IL-6 were analyzed by qRT-PCR in FLSs with PSTPIP2 overexpression. **(D)** The protein levels of TNF-α, IL-1β, and IL-6 were analyzed by Western blot in FLSs with PSTPIP2 overexpression. **(E)** The levels of TNF-α, IL-1β, and IL-6 in FLSs supernatants were detected by ELISA. The bands or images in the figure are representative in three independent experiments. Data shown are the mean ± SD from three independent experiments. Each group contains 5 rats. ^##^*P* < 0.01 versus GV230 group; ^∗∗^*P* < 0.01 versus normal group.

For exploring the changes of the inflammatory response of FLSs in the context of PSTPIP2 overexpression, overexpressed vector GV230-PSTPIP2 for rat was used to overexpress PSTPIP2 in AIA FLSs. Western blot and qRT-PCR results (Figures 2A,B) showed that the expression of PSTPIP2 was up-regulated observably in GV230-PSTPIP2 group compared with GV230 group. This indicated that the transient transfection of GV230-PSTPIP2 was successful. Western blot (Figure 2D) and qRT-PCR (Figure 2C) results showed that the protein levels of TNF-α, IL-1β, and IL-6 were reduced in GV230-PSTPIP2 group compared with GV230 group. ELISA results (Figure 2E) suggested that the levels of TNF-α, IL-1β, and IL-6 in FLSs supernatant were obviously decreasing with PSTPIP2 overexpression. These results indicated that overexpression of PSTPIP2 could suppress the inflammatory response of FLSs.

### PSTPIP2 Silencing Promotes the Inflammatory Response of FLSs

**FIGURE 3:**
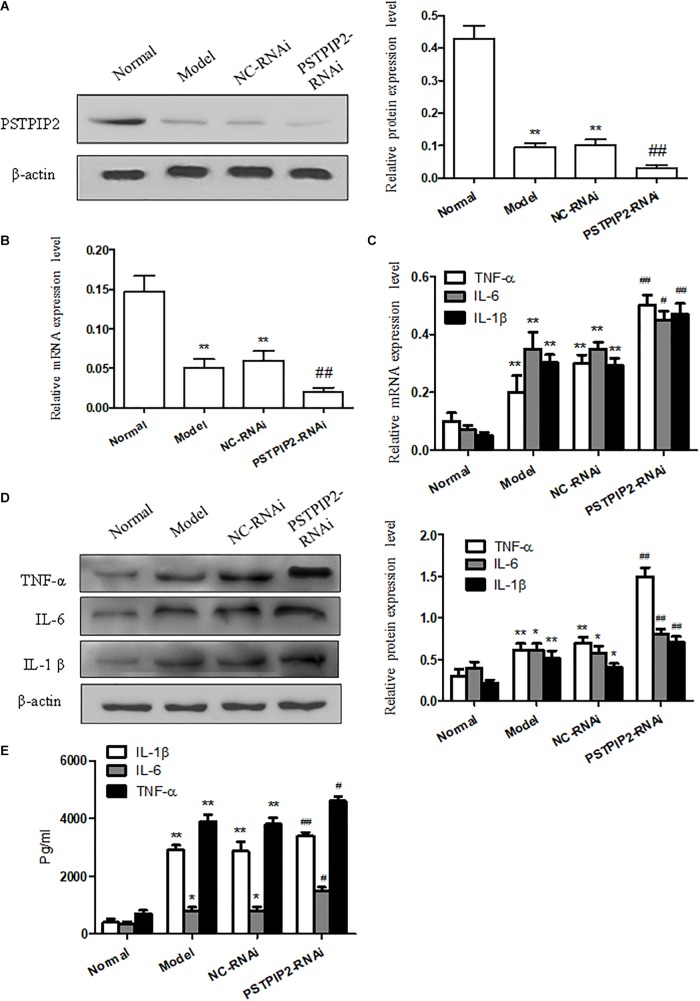
PSTPIP2 silencing promotes FLSs inflammatory response. **(A)** The protein level of PSTPIP2 was analyzed by Western blot in FLSs with PSTPIP2 silencing. **(B)** The mRNA level of PSTPIP2 was analyzed by qRT-PCR in FLSs with PSTPIP2 silencing. **(C)** The mRNA levels of TNF-α, IL-1β, and IL-6 were analyzed by qRT-PCR in FLSs with PSTPIP2 silencing. **(D)** The protein levels of TNF-α, IL-1β, and IL-6 were analyzed by Western blot in FLSs with PSTPIP2 silencing. **(E)** The levels of TNF-α, IL-1β, and IL-6 in FLSs supernatants were detected by ELISA. The bands or images in the figure are representative in three independent experiments. Each group contains 5 rats. Data shown are the mean ± SD from three independent experiments. ^#^*P* < 0.05, ^##^*P* < 0.01 versus NC-RNAi group; ^∗^*P* < 0.05, ^∗∗^*P* < 0.01 versus normal group.

To provide additional evidence to demonstrate that PSTPIP2 is involved in the inflammatory response of FLSs, the specific rat siRNA was used to knockdown the PSTPIP2 gene in AIA FLSs. The mRNA and protein levels of PSTPIP2 (Figures 3A,B) were reduced remarkably in PSTPIP2-RNAi group compared with the cells transfected with control siRNA (NC-RNAi group). This indicated that the transient transfection of PSTPIP2-RNAi was successful. Western blot (Figure 3D) and qRT-PCR (Figure 3C) results showed that the levels of TNF-α, IL-1β, and IL-6 were increased in PSTPIP2-RNAi group compared with NC-RNAi group. ELISA results (Figure 3E) suggested that the levels of TNF-α, IL-1β, and IL-6 were increased obviously in FLSs supernatant with PSTPIP2 silencing. These results indicated that PSTPIP2 silencing could promote the inflammatory response of FLSs.

### PSTPIP2 Suppresses the Proliferation of FLSs

**FIGURE 4:**
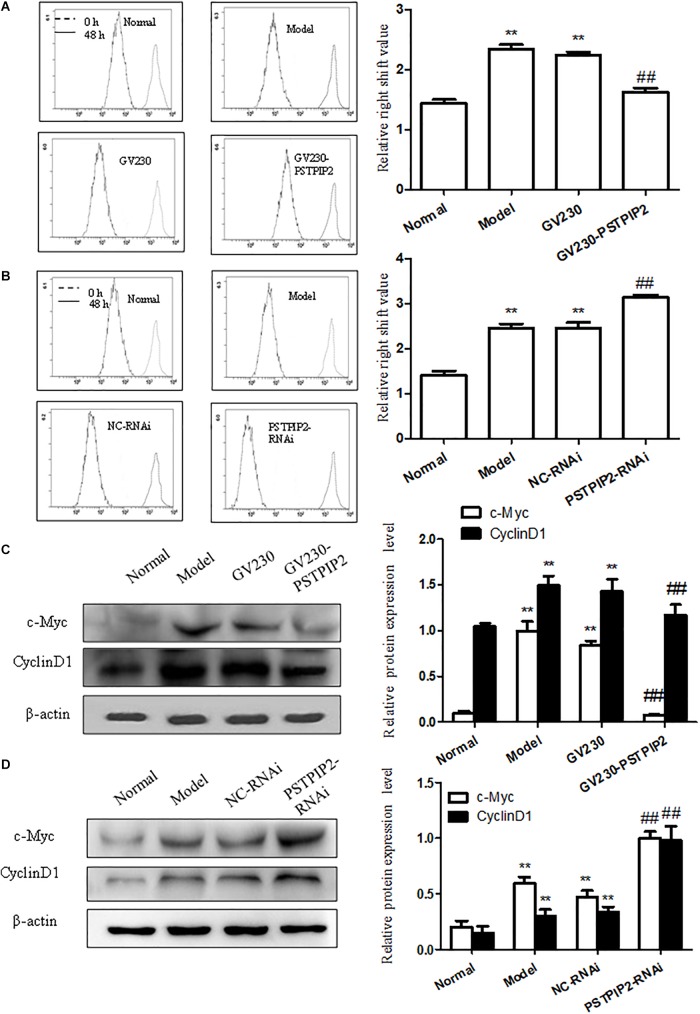
PSTPIP2 suppresses FLSs proliferation. **(A)** CFDA SE proliferation assay with PSTPIP2 overexpression. **(B)** CFDA SE proliferation assay with PSTPIP2 silencing. **(C)** The protein levels of c-Myc and Cyclin D1 were analyzed by Western blot in FLSs with PSTPIP2 overexpression. Representative bands and images of three independent experiments. **(D)** The protein levels of c-Myc and Cyclin D1 were analyzed by Western blot in FLSs with PSTPIP2 silencing. Each group contains 5 rats. The bands or images in the figure are representative in three independent experiments. Data shown are the mean ± SD from three independent experiments. ^##^*P* < 0.01 versus GV230 or NC-RNAi group; ^∗∗^*P* < 0.01 versus normal group.

To explore the role of PSTPIP2 in the proliferation of FLSs, we measured the effect of PSTPIP2 overexpression and silencing on the proliferation of FLSs. Significantly, Western blot results suggested that the expression of c-Myc and Cyclin D1 was down-regulated with PSTPIP2 overexpression (Figure 4C). CFDA SE cell proliferation assay results (Figure 4A) suggested that the proliferation of FLSs was suppressed with PSTPIP2 overexpression. Similarly, cell cycle analysis (Supplementary Figure S1F) also suggested that overexpression of PSTPIP2 resulted in a significant decrease in the proportion of cells in the S and G2/M phase. These results indicated that overexpression of PSTPIP2 could suppress the proliferation of FLSs. Lipidosome-mediated transfection resulted in PSTPIP2 knockdown in AIA FLSs. As same as expected, the expression of c-Myc and Cyclin D1 (Figure 4B) was up-regulated with PSTPIP2 silencing. CFDA SE cell proliferation assay results (Figure 4D) suggested that the proliferation of FLSs was promoted with PSTPIP2 silencing. Similarly, cell cycle analysis (Supplementary Figure S1G) suggested that treatment of FLSs with PSTPIP2 siRNA resulted in an obvious increase in the proportion of cells in the S and G2/M phase. These results indicated that PSTPIP2 silencing could promote FLSs proliferation. In conclusion, our study showed that PSTPIP2 inhibits the proliferation of FLSs.

### PSTPIP2 Modulates the Proliferation and Inflammatory Response of FLSs, Which Might Be Closely Associated With NF-κB Signaling Pathway

**FIGURE 5:**
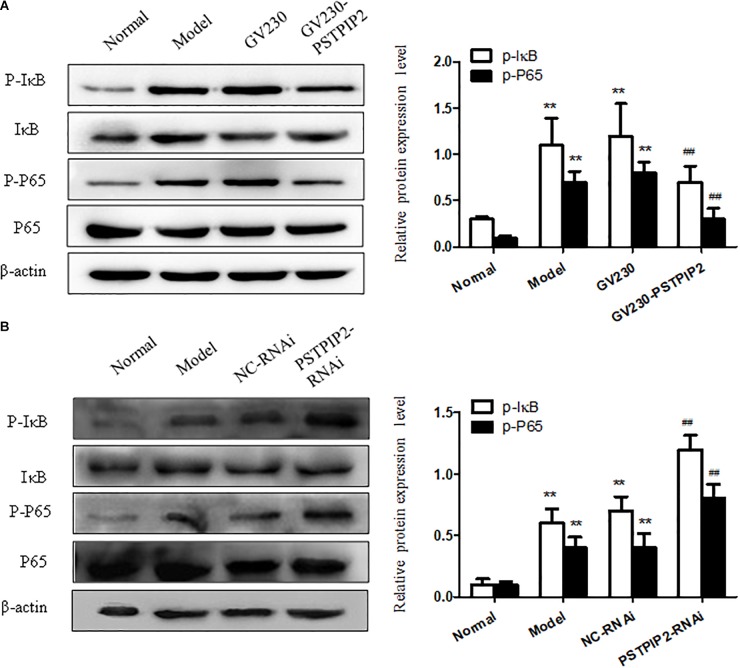
Studying of NF-κB signaling pathway. **(A)** The protein levels of p-P65 and p-IκB were detected by Western blot in FLSs with PSTPIP2 overexpression. **(B)** The protein levels of p-P65 and p-IκB were detected by Western blot in FLSs with PSTPIP2 silencing. Each group contains 5 rats. The bands or images in the figure are representative in three independent experiments. Data shown are the mean ± SD from three independent experiments. ^##^*P* < 0.01 versus GV230 or NC-RNAi group; ^∗∗^*P* < 0.01 versus normal group.

We measured the protein levels of p-P65 and p-IκB in FLSs transfected with GV230-PSTPIP2 or PSTPIP2-RNAi. Western blot results suggested that the expression of p-P65 and p-IκB were reduced with PSTPIP2 overexpression (Figure 5A) but increased with PSTPIP2 silencing (Figure 5B). These results indicated that PSTPIP2 modulates the proliferation and inflammatory response of FLSs, which might be closely associated with NF-κB signaling pathway.

## Discussion

Adjuvant-induced arthritis (AIA) is a model of experimental RA that is induced by injection of CFA (Li et al., 2014, 2016). AIA animal model has similar characteristics to RA in aspects of histology and immunology and is a useful test system for evaluating the efficiency of therapies for RA (Li et al., 2017). Therefore, AIA was chosen to study the role of PSTPIP2. Recent progress has substantiated that FLSs are the key effector cells in inflammatory arthritic diseases (Feldmann et al., 1996; Koga et al., 2011). Excessive proliferation of FLSs is one of the critical features of RA, which leads to cartilage and bone destruction (Pap et al., 2000). In addition, hyperplastic FLSs potentially promote lymphocyte and macrophage infiltration (Smeets et al., 2003), recruitment and retention by producing cytokines (Larsen et al., 2012), chemokines (Lee et al., 2013), extracellular matrix proteins (Smeets et al., 2003), and cell adhesion molecules (Park et al., 2014). Moreover, a wide range of pro-inflammatory cytokines, such as TNF-α, IL-1β, and IL-6, place a premium on RA progression and play an essential role in the inflammatory pathogenesis of RA [36]. However, there are no approved drugs are known for targeting FLSs in RA, and the underlying mechanisms of driving FLSs activation remain unresolved. Hence, targeted inhibition of the proliferation and inflammatory response of FLSs may be a potentially complement to current therapeutics.

PSTPIP2, a protein associated with auto-inflammatory disease, which is involved in macrophage activation, neutrophil motility, and osteoclast differentiation, has been recently proposed to play a role in the development of auto-inflammatory bone disorders (Drobek et al., 2015; Marzano et al., 2016). Mutations of PSTPIP2 gene are associated with the auto-inflammatory disorder chronic multifocal osteomyelitis in mice (Ferguson et al., 2006). Here in our study, to explore whether PSTPIP2 plays a role in FLSs of AIA, the expression of PSTPIP2 were detected by WB and qRT-PCR first. Surprisingly, the results indicated that the expression of PSTPIP2 was significantly down-regulated in AIA synovial tissues and FLSs compared with control group (normal group). Likewise, immunohistochemical and immunofluorescence analysis demonstrated that the expression of PSTPIP2 was also reduced in AIA synovial tissues and FLSs, respectively.

Many evidences indicated that NF-κB pathway is related to RA. It could regulate inflammatory response and cell proliferation, and it is also one of the most crucial transcription factors (Zhu et al., 2016). Moreover, the destruction of joint was alleviated with the inhibition of NF-κB signaling pathway in AIA models (Zhou et al., 2014; Wang et al., 2015). Thus, it is of great significance to study the role of NF-κB signaling in FLSs for the treatment of RA.

In our experiments, we investigated the role of PSTPIP2 in FLSs of AIA rat. We first discovered the key role of PSTPIP2 in FLSs, which is a new breakthrough in the development of PSTPIP2 research. Previous studies have shown that PSTPIP2 plays an important role in hematopoietic cells. In recent years, a growing number of researchers have discovered that PSTPIP2 play a role in inflammatory diseases. Our laboratory found differential expression of PSTPIP2 in rat synovium and FLSs, and then we successfully discovered the role of PSTPIP2 in the inflammatory response and proliferation of FLSs. However, our experiments still have certain limitations. We only explored some of the effects of PSTPIP2 on FLSs *in vitro*. The inhibitory effect of PSTPIP2 on inflammation needs to be further studied in animals and even in humans. Our experimental results indicated that PSTPIP2 inhibits the release of IL-1β. Prior to this, the relationship between PSTPIP2 and IL-1β has been reported (Lukens et al., 2014). It is still needed to further explore the relationship between PSTPIP2 and IL-1β, which is a very good entry point for studying PSTPIP2 in inflammation. In addition, our experimental results indicated that silencing or overexpression of PSTPIP2 can effectively regulate the NF-κB pathway. This may be an important mechanism by which PSTPIP2 is involved in the inflammatory response and cell proliferation. There are still many open questions, for example, whether PSTPIP2 acts directly on the NF-κB pathway in FLSs. Our data are not enough to demonstrate the causal relationship between PSTPIP2 and NF-κB. Moreover, whether the activation of NF-κB can inhibit the expression of PSTPIP2 still needs to be further studied.

Previous studies showed that PSTPIP2 inhibits the proliferation and activation of hematopoietic cells. We discovered that PSTPIP2 can inhibit the proliferation of FLSs, which is of great significance to studying the role of PSTPIP2 in RA. Our results indicated that silencing or overexpression of PSTPIP2 can effectively regulate the proliferation of FLSs. Further researches are needed on the mechanism by which PSTPIP2 inhibits the proliferation of FLSs. We conjecture that PSTPIP2 may regulate the proliferation of FLSs by altering the deformational movement of synovial cells due to PSTPIP2 is a skeletal protein.

In summary, the results of this study showed that PSTPIP2 suppresses the proliferation and inflammatory response of FLSs. Notably, transient transfection of GV230-PSTPIP2 completely reduced the level of inflammatory cytokines (TNF-α, IL-1β, and IL-6) and inhibited the proliferation of FLSs, but it is contrary with PSTPIP2 silencing. The NF-κB pathway is associated with the expression of PSTPIP2. Therefore, PSTPIP2 may be a novel therapeutic target for RA, which might be associated with the NF-κB pathway.

## Conclusion

In this study, we analyzed the effect of PSTPIP2 on the inflammatory response and proliferation of FLSs in experimental arthritis model *in vitro*. We found that the expression of PSTPIP2 was significantly reduced in synovial tissues of inflamed joints as well as in FLSs. Overexpression or silencing of PSTPIP2 was associated with the proliferation and inflammatory response of FLSs. In addition, the expression of P65 and IκB was related to the expression of PSTPIP2. These results indicated that PSTPIP2 could inhibit the inflammatory response and proliferation of FLSs in AIA, which might be closely related to NF-κB pathway. From all of the above, we believe that PSTPIP2 would be a new target for the treatment of RA, potentially providing new ideas for the study of RA.

## Author Contributions

All authors listed have made a substantial, direct and intellectual contribution to the work, and approved it for publication.

## Conflict of Interest Statement

The authors declare that the research was conducted in the absence of any commercial or financial relationships that could be construed as a potential conflict of interest. The handling Editor declared a shared affiliation, though no other collaboration, with the authors at time of review.

## Supplementary Material

Figure S1 (A) The photos of the right hind paw of Normal and Model rats on day 24. **(B)** The Arthritis score of Normal and Model rats at different time points. **(C)** The levels of TNF-α, IL-6 and IL-1β in the serum of Normal and model rats. **(D)** The paw swelling of right hind paw of Normal and Model rats at different time point. **(E)** The expression of Vimentin in the cell we cultured was detected by immunofluorescence staining analysis. **(F)** Cell cycle was examined by flow cytometry of FLSs with PSTPIP2 overexpression. **(G)** Cell cycle was examined by flow cytometry of FLSs with PSTPIP2 silencing.
