# Supplementary figures and images for: PSTPIP2 Inhibits the Inflammatory Response and Proliferation of Fibroblast-Like Synoviocytes in vitro
Source: Front Pharmacol. 2018 Dec 4;9:1432. doi: 10.3389/fphar.2018.01432 (PMC6289071; doi:10.3389/fphar.2018.01432)

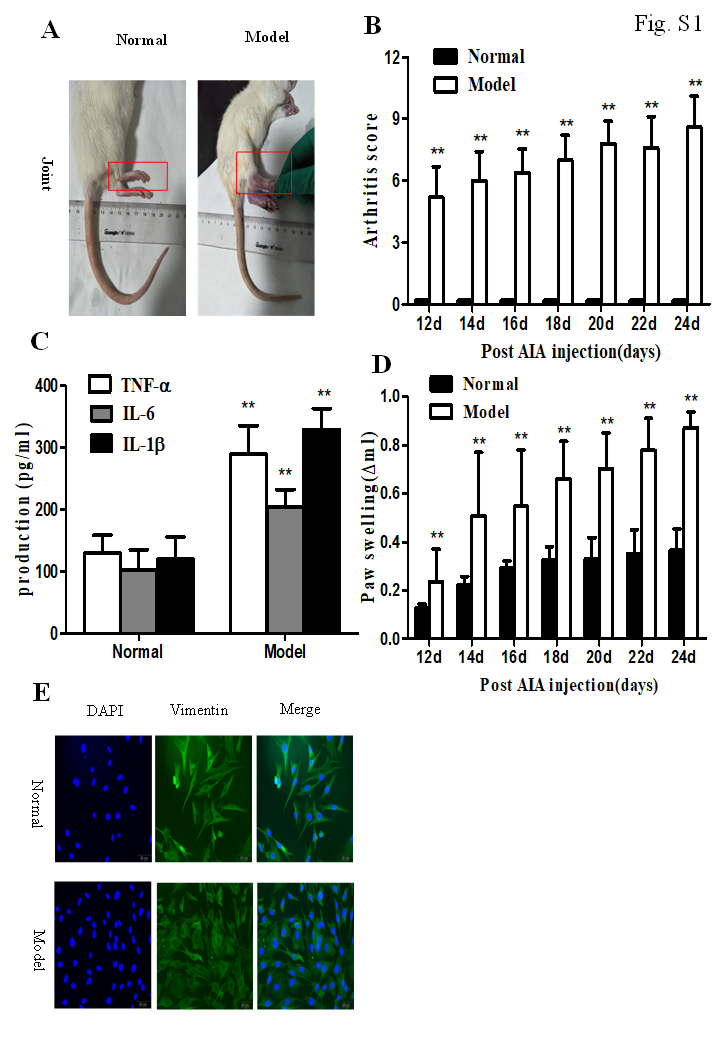

Supplement: Figure S1 — (A) The photos of the right hind paw of Normal and Model rats on day 24. (B) The Arthritis score of Normal and Model rats at different time points. (C) The levels of TNF-α, IL-6 and IL-1β in the serum of Normal and model rats. (D) The paw swelling of right hind paw of Normal and Model rats at different time point. (E) The expression of Vimentin in the cell we cultured was detected by immunofluorescence staining analysis. (F) Cell cycle was examined by flow cytometry of FLSs with PSTPIP2 overexpression. (G) Cell cycle was examined by flow cytometry of FLSs with PSTPIP2 silencing. [file Image_1.TIF]

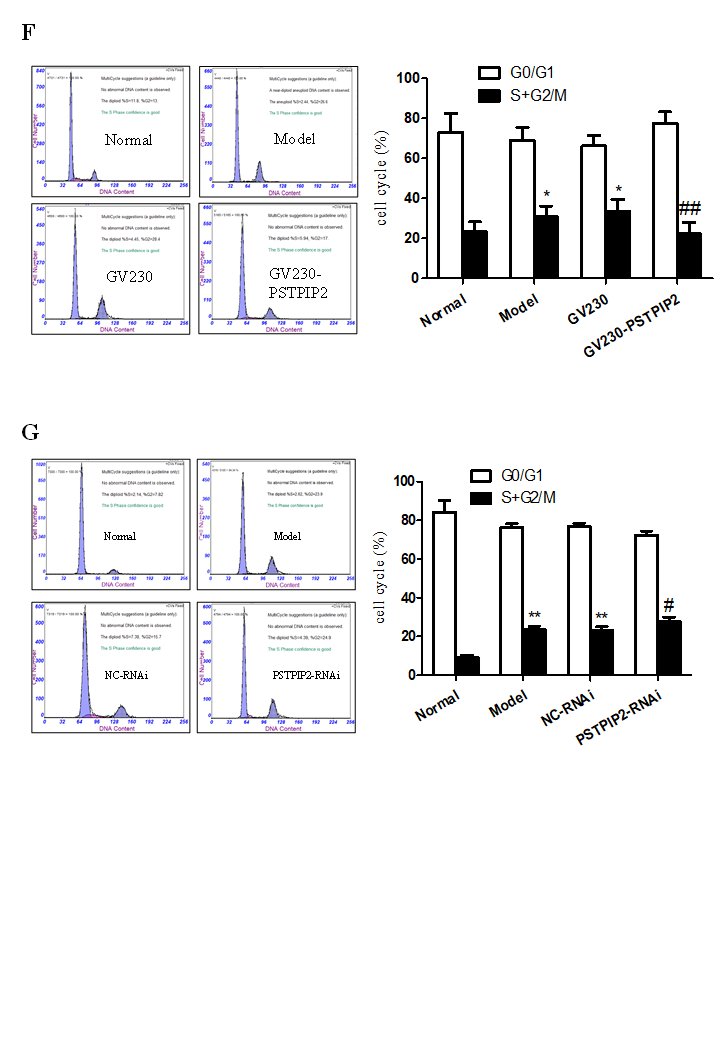

Supplement: Supplementary file 2 [file Image_2.TIF]
